# Development and validation of a measurement tool to assess student perceptions of using real patients in physical therapy education at the Rocky Mountain University, the United States: a methodological study

**DOI:** 10.3352/jeehp.2024.21.30

**Published:** 2024-11-07

**Authors:** Stacia Hall Thompson, Hina Garg, Mary Shotwell, Michelle Webb

**Affiliations:** 1 Doctor of Philosophy Program in Health Sciences, Rocky Mountain University of Health Professions, Provo, UT, USA; 2 Department of Physical Therapy, Rocky Mountain University of Health Professions, Provo, UT, USA; Hallym University, Korea

**Keywords:** Physical therapy, Students, Factor analysis

## Abstract

**Purpose:**

This study aimed to develop and validate the Student Perceptions of Real Patient Use in Physical Therapy Education (SPRP-PTE) survey to assess physical therapy student (SPT) perceptions regarding real patient use in didactic education.

**Methods:**

This cross-sectional observational study developed a 48-item survey and tested the survey on 130 SPTs. Face and content validity were determined by an expert review and content validity index (CVI). Construct validity and internal consistency reliability were determined via exploratory factor analysis (EFA) and Cronbach’s α.

**Results:**

Three main constructs were identified (value, satisfaction, and confidence), each having 4 subconstruct components (overall, cognitive, psychomotor, and affective learning). Expert review demonstrated adequate face and content validity (CVI=96%). The initial EFA of the 48-item survey revealed items with inconsistent loadings and low correlations, leading to the removal of 18 items. An EFA of the 30-item survey demonstrated 1-factor loadings of all survey constructs except satisfaction and the entire survey. All constructs had adequate internal consistency (Cronbach’s α >0.85).

**Conclusion:**

The SPRP-PTE survey provides a reliable and valid way to assess student perceptions of real patient use. Future studies are encouraged to validate the SPRP-PTE survey further.

## Graphical abstract

[Fig f2-jeehp-21-30]

## Introduction

### Background/rationale

Physical therapy education and other health professions use simulation-based learning experiences to enhance didactic knowledge, build psychomotor skills, increase confidence, and prepare students for clinical rotations [1,2]. Typical simulation-based learning experience approaches include case-based analysis, peer teaching, simulated patients (SPs), and real patients [1,2]. SPs present a higher fidelity learning experience, where trained individuals portray clinical scenarios and imitate impairments seen in clinical practice [1,2]. Despite being effective in simulation-based learning experiences, challenges persist in using SPs, such as training time, cost efficiency, replicating realistic situations or impairments, and the dynamic interplay between therapist and patient [1,2]. To combat these challenges, health professions educators have incorporated real patients with medical conditions or impairments that resemble clinical situations [2-5].

Evidence supports the use of real patients in medical education [5,6]. Experiences with real patients provide context, interest, focus, personal responsibility, complexity, different perspectives, communication practice, and a patient-centered approach to care [3-6]. Data on real patient use in physical therapy education during simulation-based learning experiences is emerging in cognitive and affective domains [3,4]. Evidence suggests that students prefer real patients, especially towards the end of their education, because real patients are predictable in terms of the skills that students are supposed to practice [2]. Therefore, information about the usefulness of real patients for physical therapy students (SPTs) in physical therapy education is lacking. More information is needed regarding the advantages and hurdles of using real patients for skill development in cognitive, affective, and psychomotor learning domains and how students perceive this educational modality.

Additionally, in simulation-based learning experiences with real patients, students may practice skills on individuals who are not injured or have no specific disease or functional limitation. Using real patients may benefit SPTs’ learning by providing opportunities to recognize how normal and abnormal physical and behavioral impairments present. Ultimately, simulation-based learning experiences with real patients may help SPTs prepare for clinical rotations and future practice.

### Objectives

This study aims to develop and validate the Student’s Perceptions on the Use of Real Patients in Physical Therapy Education (SPRP-PTE) survey tool to assess SPTs’ perceptions regarding the value, satisfaction, and confidence of learning with real patients in simulation-based learning experiences in multiple learning domains (cognitive, psychomotor, and affective).

## Methods

### Ethics statement

This study was approved by the Rocky Mountain University Institutional Review Board (Protocol #2022-249). All survey participants provided informed consent before participation, data were deidentified before review, and data storage complied with university guidelines for security and protection.

### Study design

This was a cross-sectional assessment validation study.

### Setting

**Fig. 1.**
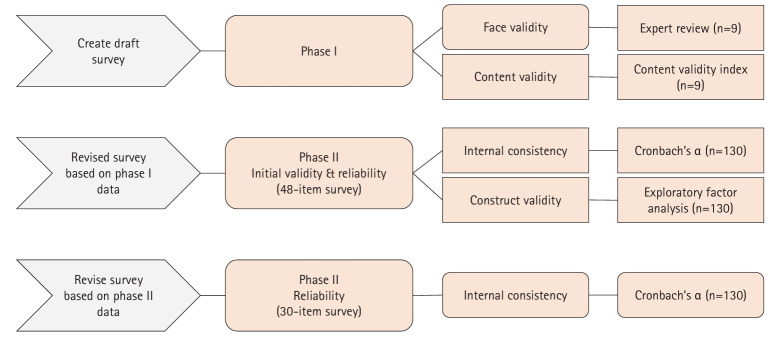
Survey flow.

This cross-sectional study was conducted in 2 phases: phase I, which involved the development and content validation of the SPRP-PTE, and phase II, which investigated its psychometric properties (Fig. 1).

### Phase I: SPRP-PTE development and content validation

#### Survey development and draft

The SPRP-PTE combined survey questions from prior research seeking SPTs’ perceptions about value, satisfaction, and confidence using SPs [2,7] and adapted based on synthesizing the literature related to real patient use in health professions education. Supplement 1 provides permission to use the survey questions [2,7]. Analysis of the SPRP-PTE draft survey revealed 3 primary constructs: value, satisfaction, and confidence, and 4 subconstructs: overall, cognitive, psychomotor, and affective learning [8]. The SPRP-PTE survey queries SPTs’ perceptions about using real patients through 4 sections with 48 construct and 8 demographic questions, using a 4-point Likert scale rating level of agreement for response selection. A matrix layout was used due to the number of constructs. The 4-point Likert scale enabled every item to be totaled, rating the level of agreement of each item, with higher scores indicating higher levels of agreement.

#### Subjects

Twelve physical therapy educators from the primary author’s professional network were recruited for expert review based on their knowledge and experience regarding survey research, PTE, and rehabilitation education practices [9-12].

#### Procedures

Experts received an email with a link to the web-based questionnaire provided by Qualtrics XM (https://www.qualtrics.com/). Experts were given background information on the survey’s purpose and definitions of constructs and asked to rate agreement as “yes” or “no” on 7 questions for face validity. Expert reviewers also rated each survey question’s relevance and necessity on a 0–4 scale (irrelevant to relevant with no adjustments needed) [9,10]. Experts provided open-ended responses to clarify their ratings.

#### Data analysis

For face validity, percent agreement among experts per question was calculated by totaling the “yes” responses, dividing by the total responses, and multiplying by 100 [9]. The criteria for retaining items without revision was >0.91 [9]. The content validity index (CVI) was used to calculate the level of agreement among experts to determine content validity [10,11]. The criterion to retain individual items without revision was >0.83 [11]. The CVI criterion for the entire survey was 0.90 or greater [11].

#### Outcomes

Nine physical therapy educators responded to the questionnaire (response rate, 75%). Table 1 describes the expert reviewer demographics. One hundred percent agreement was achieved on construct-related questions, and 66%–100% agreement on format-related questions for face validity (Table 2). Even though some item ratings reached a level requiring revision (<91%), open-ended comments revealed formatting concerns versus content as the basis for the level of agreement (Table 2).

Table 3 shows CVI results. The CVI criterion was met for construct-related questions (100%) and the entire survey (96%). Percent agreement varied for demographic questions.

No changes were made to construct-related questions based on the satisfactory percent agreement for face validity and CVI. However, revisions occurred to provide more explicit construct definitions, note divisions between sections in the matrices, and clarify demographic questions based on reviewer feedback. The revised survey was sent to SPTs for phase II (Supplement 2).

### Phase II: Investigation of the psychometric properties of the SPRP-PTE

#### Subjects

Survey participants included SPTs with real patient experience in PTE. Survey responses were reviewed for missing data points, removing responses if any or all matrices were unanswered or if there was less than 60% survey completion [12]. The authors used an 80% completion rate (i.e., participants answering at least 40 of 48 construct questions) as the benchmark for inclusion [12]. No data imputation was performed since the measure was newly developed. Sample adequacy was determined by ensuring a sample of 100–200 participants [13]. After data review, 130 out of 214 SPTs completed the SPRP-PTE survey, with a 60.7% completion rate (Dataset 1). Table 4 provides participant demographics.

#### Procedures

A recruitment flyer with a link to the survey was shared with program directors at accredited physical therapy programs in the United States (n=273 and student population n=37,306), posted on an SPT-specific social media page, and distributed to the authors’ known physical therapy education contacts. The SPRP-PTE survey was delivered electronically using Qualtrics. It was open for 2 months (February to April 2023), with reminder emails and posts sent every 2–3 weeks.

#### Data analysis

Exploratory factor analysis (EFA) was conducted using Intellectus Statistics 2019 (https://www.intellectusstatistics.com/). One- to 3-factor solutions were examined for each set of matrices (overall, cognitive, psychomotor, and affective learning) related to the primary survey constructs (value, satisfaction, and confidence). The Kaiser criterion with varimax rotation was used for rotation, assuming the factors would be correlated. Data were extracted using principal component analysis. The Kaiser criterion using the eigenvalue was used to determine the number of factors [12]. Factor adequacy was established by discarding loadings on multiple factors and ensuring internal consistency of 0.70 or greater [12]. Item-to-item analysis using Spearman correlation was used to determine the relationship between items in each matrix section. Items with coefficients less than 0.40 were considered for removal [12].

Sections of each matrix were utilized to assess the intended constructs, and the entire matrix was examined using Cronbach’s α. A criterion of 0.80 or better indicated adequate internal consistency [12].

## Results

### Exploratory factor analysis

For the revised 48-item SPRP-PTE survey, the EFA suggested item loading onto 9 factors (Supplement 3). Each item was individually loaded on as many as 3 factors. The initial Cronbach’s α was 0.97. Because the SPRP-PTE survey measured multiple subconstructs, EFA was completed on each matrix (overall learning, cognitive, psychomotor, and affective) to determine individual subconstruct validity. Item-to-item and item-to-total analyses were completed if factor loadings did not meet the criterion.

The overall learning matrix contained 15 of the 48 SPRP-PTE questions. The initial EFA suggested 3 factors, with items loading onto 1–2 factors and a Cronbach’s α of 0.92. Supplement 4 provides the overall learning matrix EFA and item analysis by construct. After review, items 2 and 4 were removed, resulting in an EFA with 1-factor loading, explaining 52.79% of the variance, and Cronbach’s α of 0.90 for the 9 items.

The cognitive matrix included 12 items. The initial EFA for the cognitive matrix revealed items loading onto 2 factors with an initial Cronbach’s α of 0.92. Supplement 5 provides the cognitive matrix EFA and item analysis by construct. After review, items 3 and 4 were removed, resulting in an EFA with a 1-factor loading, explaining 67.30% of the variance, and Cronbach’s α of 0.91 for the 6 items.

Nine items were in the psychomotor matrix. The initial EFA for the psychomotor matrix revealed a 1-factor solution explaining 65.16% of the variance, and Cronbach’s α was 0.94. No changes were made to the 9-item psychomotor matrix (Supplement 6).

Twelve items were included in the affective matrix. The initial EFA revealed 2 factors and a Cronbach’s α of 0.92. Supplement 7 provides the affective matrix EFA and item analysis by construct. After analysis, items 2 and 4 were removed, and an EFA revealed a 1-factor solution explaining 69.69% of the variance with a Cronbach’s α of 0.93 for the 6 items.

#### Revised survey exploratory factor analysis

The revised SPRP-PTE survey included 30 items (Supplement 8). Each matrix assessed 1 factor believed to be related to the matrix theme of overall, cognitive, psychomotor, and affective learning. An EFA of the revised SPRP-PTE survey revealed 5 factors (Table 5). Supplement 9 contains survey results on Cronbach’s α, EFA loadings, extraneous factor loadings, and the CVI.

#### Construct exploratory factor analysis

EFA and Cronbach’s α were run for individual construct categories of value, satisfaction, and confidence, using the revised 30-item SPRP-PTE survey to examine the constructs further (Table 6).

#### Post-hoc analysis

A post-hoc analysis was conducted to analyze the relationship between the number of real patient experiences and the primary constructs of value, satisfaction, and confidence. Pearson correlations between the number of experiences and each construct (value, satisfaction, and confidence) revealed no significant findings. Linear regression of the number of experiences did not explain a significant proportion of the variation in the totals of constructs. However, positive correlations existed between all construct variables, further supporting the internal validity of the SPRP-PTE (Table 7).

### Internal consistency

The SPRP-PTE survey’s 48- and 30-item versions and each matrix displayed excellent internal consistency (Table 8).

## Discussion

### Interpretation

This study developed and validated a novel survey exploring the SPTs’ perceptions on using real patients in simulation-based learning experiences during PTE. The revised 30-item SPRP-PTE survey demonstrated face, content, and construct validity and internal consistency. Individual matrix sections (overall learning, cognitive, psychomotor, and affective) and the value and confidence constructs also demonstrated construct validity and internal consistency. Therefore, the authors suggest the 30-item SPRP-PTE survey is best interpreted by construct to measure SPT perceptions of working with real patients during simulation-based learning experiences in PTE. Faculty may use the SPRP-PTE survey to gather feedback and enhance teaching.

Prior tools assessing student perceptions of learning using SPs or real patients in simulation-based learning experiences in physical therapy education and medical education typically measure a singular construct [6,8]. Tools assessing student learning have focused on 1 learning domain [3,4,6]. The SPRP-PTE survey, therefore, is unique because it measures 7 constructs, affording a comprehensive assessment of student perceptions regarding learning and the worth of simulation-based learning experiences using real patients. As new and innovative teaching methods emerge, accurately and comprehensively assessing SPTs’ perceptions of learning methods is essential. In addition, having a tool that assesses simulation-based learning experiences with real patients throughout various learning domains is informative to faculty for experiential design and ensures that learning objectives are met.

### Limitations

This study had a sufficient sample size; however, a larger sample would be beneficial to address the survey’s ability to assess the intended constructs. The survey was lengthy, requiring an estimated 10–15 minutes to complete, possibly attributing to the 40% loss rate due to incomplete surveys.

Survey respondents ranged in schooling levels, with the majority being in the second year of PTE. Although students were at various levels in their educational journey, literature shows that students improve their confidence as they develop their skills, especially when working with real patients [2,6,7]. Future studies may compare the effects of SPs versus real patients to determine further the impact of perceived learning at various points within PTE.

Face validity was determined using expert reviewers from the author's known network, which could have led to bias toward the measure. Future studies could revisit the face validity and include individuals across medical professions outside the author’s known network. Although the survey established internal consistency after deployment, internal consistency assessment was not performed prior, thus limiting knowledge of the survey’s ability to assess the constructs consistently [13]. Other forms of reliability and validity, including concurrent or predictive validity or test-retest reliability, were not evaluated, which might have further strengthened the survey; however, they were deferred due to the significant number of changes to the 48-item SPRP-PTE. Future studies may address additional validity and reliability results to strengthen the psychometric properties of the SPRP-PTE.

Lastly, this is the first time the SPRP-PTE survey was distributed, and the initial EFA prompted a reduction in the number of items. Due to the number of items removed, the authors conducted a second round of EFA instead of confirmatory factor analysis (CFA). CFA was not completed for several reasons: (1) the SRP-PTE was a new measure that warranted an understanding of item relationship to each other without an expectation about the number of common factors being measured [14]; (2) the 18-item reduction essentially created a new tool; therefore, factors must be identified before confirming a factor structure [12,14]; (3) the survey had a matrix structure, indicating that items may cross-load and necessitating a better understanding of the factors before conducting CFA [15]; and (4) the literature suggests that CFAs should be conducted with a new sample ideally [15], which will occur in a subsequent study.

### Generalizability

As the SPRP-PTE survey can measure multiple constructs surrounding learning in PTE, the tool may also measure SPTs’ perceptions of learning using different pedagogical methods in simulation-based learning experiences, such as virtual reality and telehealth activities. Simulation-based learning experience activities can range in fidelity levels; therefore, ascertaining student perceptions of multiple aspects of learning beyond real patients is vital. Although it was assessed with SPTs, this survey could be utilized for other medical students participating in simulation-based learning experiences using real patients during didactic education, as the language is neutral and not discipline-specific. For example, occupational therapy and speech-language pathology programs could apply this survey to their real patient’s experiences in didactics to assess student perceptions, as they are also rehabilitative professions. Lastly, as the SPTs and various programs were from across the United States, the results can be applied throughout PTE.

### Conclusion

This study provides a novel, valid tool for assessing simulation-based learning experiences with real patients, addressing concerns about future research in simulation-based learning experiences [15]. Our research established the SPRP-PTE’s face, content, and construct validity. We suggest future studies confirm these results and add additional validity and reliability factors across allied health populations to strengthen the tool.

## Figures and Tables

**Figure f2-jeehp-21-30:**
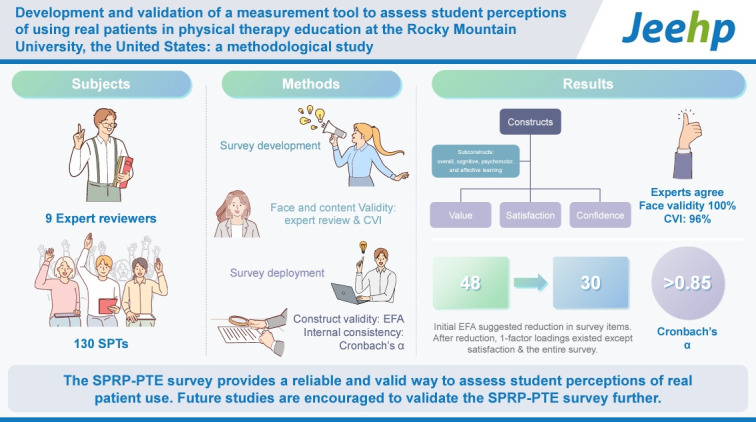


**Table 1.** Expert reviewer demographics

Expert reviewer	Years of experience as a physical therapy educator	Years of experience as a researcher	Years of experience as a clinician
1	<1	1–5	6–10
2	<1	1–5	6–10
3	1–5	1–5	11–15
4	1–5	1–5	16–20
5	1–5	1–5	16–20
6	6–10	6–10	0
7	6–10	1–5	16–20
8	6–10	6–10	25–30
9	11–15	6–10	25–30

**Table 2.** Face validity results

Question no.	Question text	Agreement %	Comments
1	The layout of the survey is effective.	77.78	Demarcations needed
2	The matrix format aids in decreasing the time to complete the survey.	100.00	
3	Grammar is appropriate for the intended audience. Correct spelling and sentence construction are present.	88.89	Provide definitions
4	Instructions are clear for each question.	77.78	Provide definitions
5	Items are structured in a well-thought-out layout and progression.	66.67	Need clear distinction between sections
6	Items are reasonable in relation to the purpose of the survey.	100.00	
7	At its face value, do you believe this survey measures the 3 constructs in the context of the 3 learning domains?	100.00	

**Table 3.** Content validity index results

Survey question	CVI (%)	Reviewer comments
Enrolled in a CAPTE-accredited institution	100.00	
Region of the United States where the program is located.	77.77	- Not sure that’s relevant to your aims.
		- For ‘other,’ there are physical therapy students worldwide; consider adding an option for what country they are from.
Have you had experience with real patients?	100.00	
Indicate the number of real patient experiences.	88.88	- Consider using ranges.
		- What does experience mean? How should students count experiences?
		- Maybe use contact hours?
		- Maybe consider a definition for experiences.
Overall learning matrix	100.00	
Cognitive domain matrix	100.00	
Psychomotor domain matrix	100.00	
Affective domain matrix	100.00	
Classification of institution (public/private)	88.88	- Class size?
Year in physical therapy school	100.00	
What is your age?	100.00	
Which classes did you have experiences with real patients?	100.00	- May want to use “typically”
		- Would recommend further clarification of normal.
Would you be willing to participate in a semi-structured interview?	100.00	

CVI, content validity index; CAPTE, Commission on Accreditation in Physical Therapy Education.

**Table 4.** Participant demographics

Variable	Value
Region	
South Atlantic	38 (29.23)
Middle Atlantic	15 (11.54)
East North Central	21 (16.15)
West North Central	13 (10.00)
West South Central	15 (11.54)
New England	9 (6.92)
Pacific	1 (0.77)
East South Central	0
Mountain	18 (13.85)
No. of real patient experiences	28.20±50.4 (1–320)
Year in physical therapy school	
1st year	34 (26.15)
2nd year	58 (44.62)
3rd year	28 (29.23)
Type of university	
Public	44 (33.85)
Private	83 (63.85)
Unknown	3 (2.31)
Age (yr)	24.60±2.3 (21–36)

Values are presented as number (%) or mean±standard deviation (range).

**Table 5.** 30-item revised survey exploratory factor analysis

Variable	Psychomotor	Cognitive	Affective	Overall learning	Mixed	Communality
Variance (%)	20.62	14.57	14.62	14.76	5.61	
1				-0.44	0.64	0.69
2				-0.73		0.68
3		-0.34				0.27
4				-0.60	0.47	0.68
5				-0.69		0.59
6				-0.66		0.62
7	-0.36			-0.60		0.56
8				-0.83		0.86
9	-0.37			-0.59		0.67
10		-0.84				0.72
11		-0.74				0.68
12		-0.74				0.74
13		-0.85				0.75
14		-0.67				0.63
15	-0.40	-0.71				0.76
16	-0.56				0.55	0.68
17	-0.68					0.66
18	-0.74					0.75
19	-0.63					0.61
20	-0.77					0.80
21	-0.79					0.73
22	-0.80					0.80
23	-0.81					0.77
24	-0.90					0.96
25			0.70			0.60
26			0.69			0.69
27			0.84			0.82
28			0.72	-0.33		0.73
29			0.85			0.82
30			0.73	-0.32		0.72

**Table 6.** Construct exploratory factor analysis

Variable	Value (1 factor)	Satisfaction (factor 1)	Satisfaction (factor 2)	Confidence (1 factor)
Variance (%)	38.96	31.54	23.85	50.12
Cronbach’s α	0.85	0.88		0.89
1	-0.65	-0.34	-0.61	-0.74
2	-0.59		-0.82	-0.73
3	-0.43		-0.90	-0.78
4	-0.44	-0.52	-0.42	-0.62
5	-0.62	-0.38		-0.72
6	-0.68	-0.71		-0.73
7	-0.74	-0.85		-0.74
8	-0.74	-0.80		-0.74
9	-0.58	-0.60		-0.61
10	-0.68	-0.57	-0.33	-0.67

**Table 7.** Correlations between constructs and number of real patient experiences

Combination	r^2^	95% CI	No.	P-value
No. of experiences–total value	0.03	-0.15 to 0.20	129	0.764
No. of experiences–total satisfaction	0.10	-0.07 to 0.27	129	0.574
No. of experiences–total confidence	0.12	-0.06 to 0.28	129	0.574
Total value–total satisfaction	0.88	0.84 to 0.92	129	<0.001
Total value–total confidence	0.81	0.74 to 0.86	129	<0.001
Total satisfaction–total confidence	0.86	0.81 to 0.90	129	<0.001

CI, confidence interval.

**Table 8.** Internal consistency results

	Initial Cronbach’s α (original 48-item survey)	Final Cronbach’s α (final 30-item survey)
Survey (entire survey)	0.97	0.94
Overall learning matrix	0.91	0.90
Cognitive matrix	0.92	0.91
Psychomotor matrix	0.94	0.94
Affective matrix	0.92	0.93
Value	0.89	0.85
Satisfaction	0.91	0.88
Confidence	0.92	0.89

## Footnotes

**Data availability**
Data files are available from Harvard Dataverse: https://doi.org/10.7910/DVN/F5POU5 Dataset 1. One hundred thirty students’ responses to the student’s perceptions on the use of real patients in physical therapy education (SPRP-PTE) for exploratory factor analysis and reliability testing.

## Supplementary materials

Supplementary files are available from Harvard Dataverse: https://doi.org/10.7910/DVN/F5POU5

Supplement 1. Permission to use surveys.

Supplement 2. 48-item survey used for psychometric property evaluation.

Supplement 3. Revised 48-item survey exploratory factor analysis loading.

Supplement 4. Exploratory factor analysis results for the overall learning matrix.

Supplement 5. Exploratory factor analysis results for the cognitive matrix.

Supplement 6. Exploratory factor analysis results for the psychomotor matrix.

Supplement 7. Exploratory factor analysis results for the affective matrix.

Supplement 8. Revised survey matrices (30-item survey).

Supplement 9. 30-item survey final results, including exploratory factor analysis, content validity index, Cronbach’s α.

Supplement 10. Audio recording of the abstract.
