# Supplementary material for: Development and validation of a measurement tool to assess student perceptions of using real patients in physical therapy education at the Rocky Mountain University, the United States: a methodological study
Source: J Educ Eval Health Prof. 2024 Nov 7;21:30. doi: 10.3352/jeehp.2024.21.30 (PMC11637597; doi:10.3352/jeehp.2024.21.30)
Supplement: Supplementary file 2 — Supplement 1. Permission to use surveys. [file jeehp-21-30-suppl1.pdf]

Rocky Mountain University

Stacia Thompson <stacia.britton@rm.edu>

Study Questionnaire

Beth Black <bblack@oakland.edu> Thu, Mar 17, 2022 at 3:11 PM
To: Stacia Britton <stacia.britton@rm.edu>

This Message originated outside your organization.

HI Stacia,
Given the length of time since the study publication, I am so sorry that I no longer have a copy of the original questionnaire but we used a 10-cm visual analog scale with question-appropriate anchor comments at each end of each scale (for example: not at all useful at one end and very useful at the other end) as well as an open comment section after each one of the questions that are in Table 5 of the study. Please feel free to use or adapt for your study. Best of luck!
Beth
[Quoted text hidden]
--
Beth Black, PT, DSc
Clinical Associate Professor
Physical Therapy Program
School of Health Sciences
Oakland University
Rochester, Michigan

Rocky Mountain University

Stacia Thompson <stacia.britton@rm.edu>

Study Questionnaire

Edward Giesbrecht <Ed.Giesbrecht@umanitoba.ca> Sun, Mar 20, 2022 at 7:30 PM
To: Stacia Britton <stacia.britton@rm.edu>

This Message originated outside your organization.

Dear Stacia:

Thank you for your email. Yes, you are welcome to use the survey and adapt it as needed, citing the original source. I have attached a copy of the survey.

Good luck with your project.

Regards

Ed Giesbrecht

From: Stacia Britton <stacia.britton@rm.edu>
Date: Sunday, March 20, 2022 at 5:14 PM
To: "ed.giesbrecht@med.umanitoba.ca" <ed.giesbrecht@med.umanitoba.ca>
Subject: Fwd: Study Questionnaire

Caution: This message was sent from outside the University of Manitoba.

[Quoted text hidden]

Blank copy of survey.pdf
64K
