# Supplementary material for: Development and validation of a measurement tool to assess student perceptions of using real patients in physical therapy education at the Rocky Mountain University, the United States: a methodological study
Source: J Educ Eval Health Prof. 2024 Nov 7;21:30. doi: 10.3352/jeehp.2024.21.30 (PMC11637597; doi:10.3352/jeehp.2024.21.30)
Supplement: Supplementary file 3 — Supplement 2. 48-item survey used for psychometric property evaluation. [file jeehp-21-30-suppl2.docx]

**Supplement 2.** 48-item survey used for psychometric property evaluation

Physical therapy student perceptions on the use of real patients in physical therapy education

Start of block: introduction and consent

**Q1** Informed consent

Dear doctor of physical therapy student,

I am inviting you to take part in a research project that is part of my degree requirements. Before you decide to be in this project, it is important that you understand why the project is being done and what it will involve. Please take the time to read the following information carefully. Please contact me if there is anything that is not clear or if you need more information.

The purpose of this project is to determine the perceptions of physical therapy students on the use of real patients in the classroom setting for learning physical therapy skills. This research has 2 components. I am asking you to participate in the first component, a survey. I ask you to fill out the survey to the best of your abilities with specific information regarding your perceptions on learning physical therapist skills using real patients. The estimated time to complete the survey is 10–20 minutes. You are free to skip any question in the survey if you choose. Your identity and information will be anonymous. Your name or other identifiers will not be collected. Members of my committee and I will review the collected data.

This project will gather information to understand the best experiential learning activities that foster the development of physical therapist skills. Knowledge gained from this survey may aid physical therapy faculty in classroom activity design. I will deliver the survey via a password-protected email and store all data on a password-protected computer. I will provide the survey through Qualtrics XM, a company not affiliated with Rocky Mountain University and with its own privacy and security policies that you can find on its website: https://www.qualtrics.com/

terms-of-service/, https://www.qualtrics.com/privacy-statement/, and https://www.qualtrics.com/

security-statement/. Despite best efforts, no method of transmission over the Internet or method of electronic storage is perfectly secure or private. I cannot guarantee absolute security.

Your participation in this project is voluntary. If you decide not to participate in this research, this decision will not affect your relationship with myself, your academic institution, the sponsoring facility, course grade, or academic standing within your physical therapy program. It is up to you to decide whether or not to be in this project. If you choose to be in this project, you are free to stop at any time without giving a reason.

Thank you for considering being in this project.

Sincerely,

Stacia Hall Thompson, PT, DPT

Doctor of Philosophy student at Rocky Mountain University of Health Professions

828-244-3064

Stacia.britton@rm.edu

• I consent

• I do not consent

Skip to: end of survey if dear doctor of physical therapy student, I am inviting you to take part in a research project that... = I do not consent.

Definitions for reference for the survey

• **Real patient:** A real patient is a person who has an impairment due to natural causes or an individual without impairments presenting typically. Real patients are not simulating any portion of the impairment or deficit.

• **Cognitive skills:** Facts and knowledge needed to perform physical therapist duties.

• **Psychomotor skills:** Hands-on, performance-based skills, including physical movement, coordination, and use of motor skill(s).

• **Affective/social skills:** Attitudes, values, beliefs, opinions, interests, and motivational attributes.

• **Value:** Something that is held in high importance or worth.

• **Satisfaction:** Fulfillment of wishes or needs.

• **Confidence:** The feeling of self-assurance of one’s abilities.

End of block: introduction and consent | start of block: inclusion/exclusion

**Q2** Are you currently a student in a Commission on Accreditation in Physical Therapy Education (CAPTE)-accredited physical therapy program?

O Yes

O No

Skip to: End of survey If are you currently a student in a CAPTE-accredited physical therapy program? = No

**Q3** In what region of the country is your physical therapy program located?

O South Atlantic (DE, DC, FL, GA, MD, NC, PR, SC, VA, WV)

O Middle Atlantic (NJ, NY, PA)

O East North Central (IL, IN, MI, OH, WI)

O West North Central (IA< KS< MN, MO, NE, ND, SD)

O West South Central (AR, LA, OK, TX)

O New England (CT, ME, MA, NH, RI, VT

O Pacific (AK, CA, HI, OR, WA)

O East South Central (AL, KY, MS, TN)

O Mountain (AZ, CO, ID, MT, NV, NM, UT, WY)

O Other (outside US: Canada, Scotland)

Skip to: End of survey If in what region of the country is your physical therapy program located? = Other (outside US: Canada, Scotland)

**Q4** Have you had experiences with real patients during your physical therapy didactic curriculum?

(A real patient is a person who has an impairment due to natural causes or an individual without impairments presenting typically. Real patients are not simulating any portion of the impairment or deficit.)

O Yes

O No

Skip to: End of survey If have you had experiences with real patients during your physical therapy didactic curriculum? (A... = No

Skip to: Q16 If Have you had experiences with real patients during your physical therapy didactic curriculum? (A... = Yes

**Q5** To the best of your recollection, please indicate the number of sessions/experiences with real patients (during your didactic education) that you have had. Please count multiple sessions with the same patient as a separate session/experience, no matter the length of time spent with the real patient. For example, 1 test performed at one time and an evaluation performed at another would count as 2 experiences.

▼ 1 ... 21+

*Display this question: If to the best of your recollection, please indicate the number of sessions/experiences with real pa... = 21+*

**Q5a** If greater than 21 sessions/experiences, please estimate the number of sessions/experiences in the text box below.

**End of block: inclusion/exclusion questions | start of block: block 8**

**Matrix questions**

**Overall learning matrix**


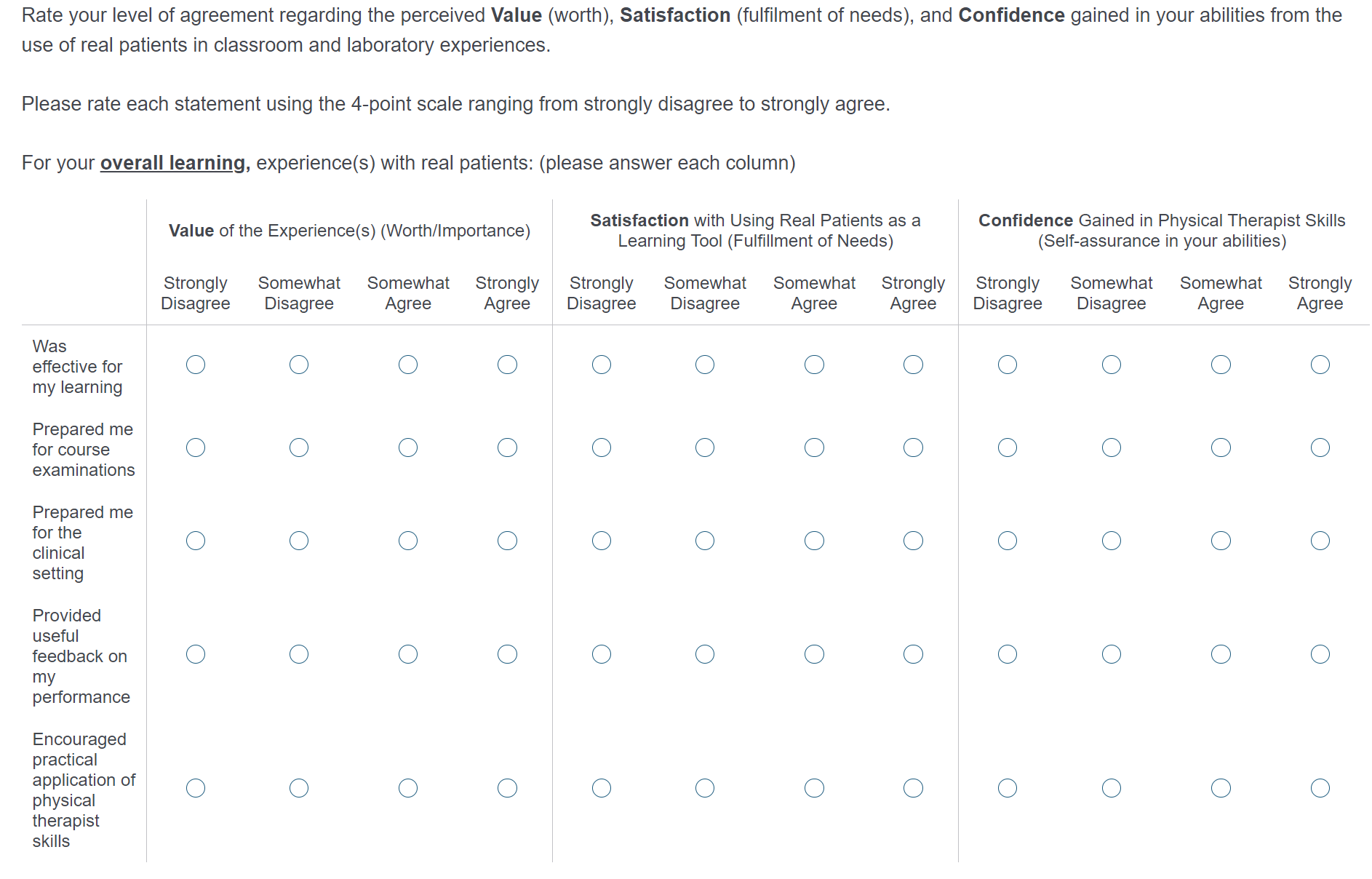


**Cognitive matrix**


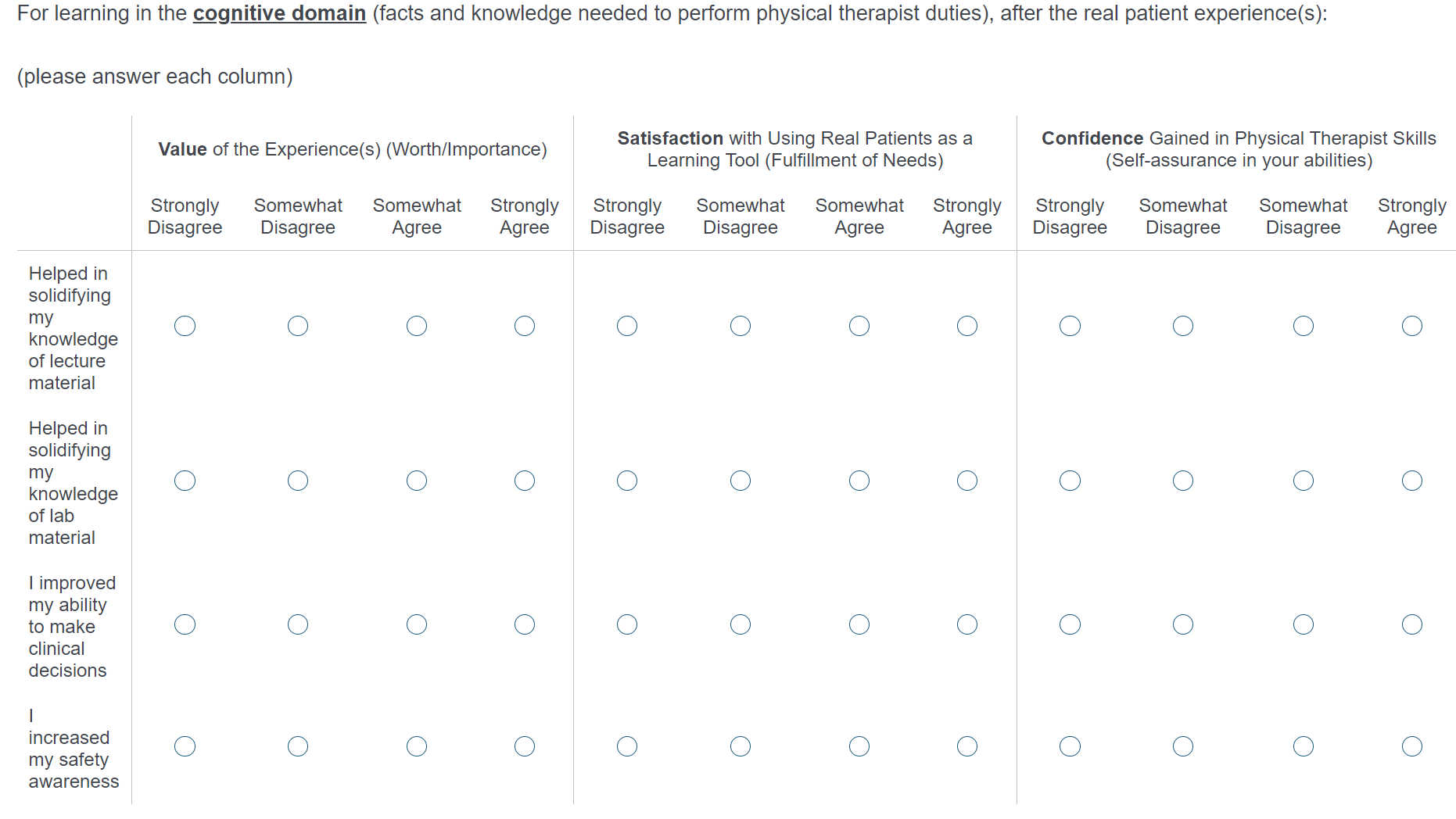


**Psychomotor matrix**
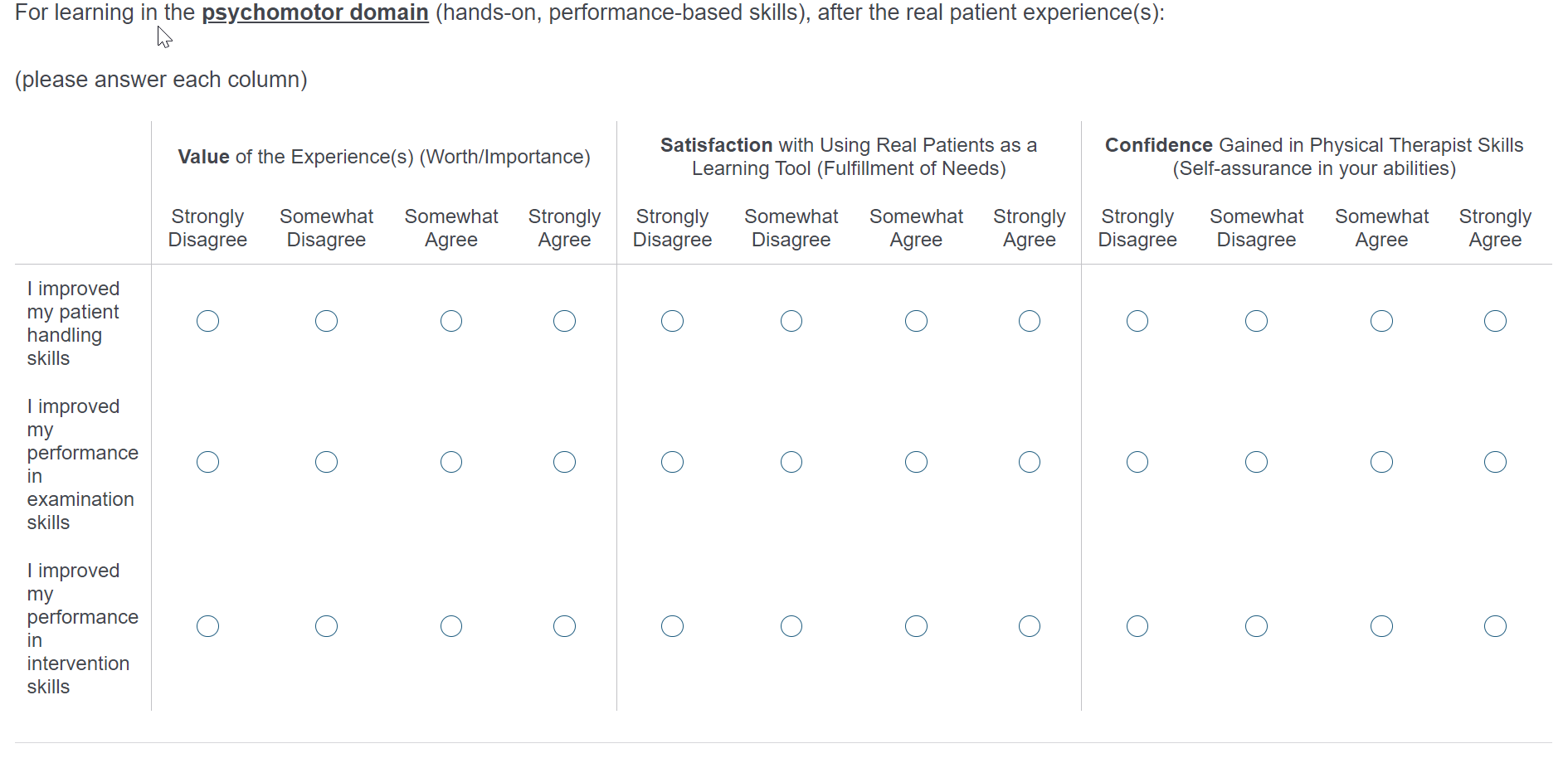


**Affective matrix**
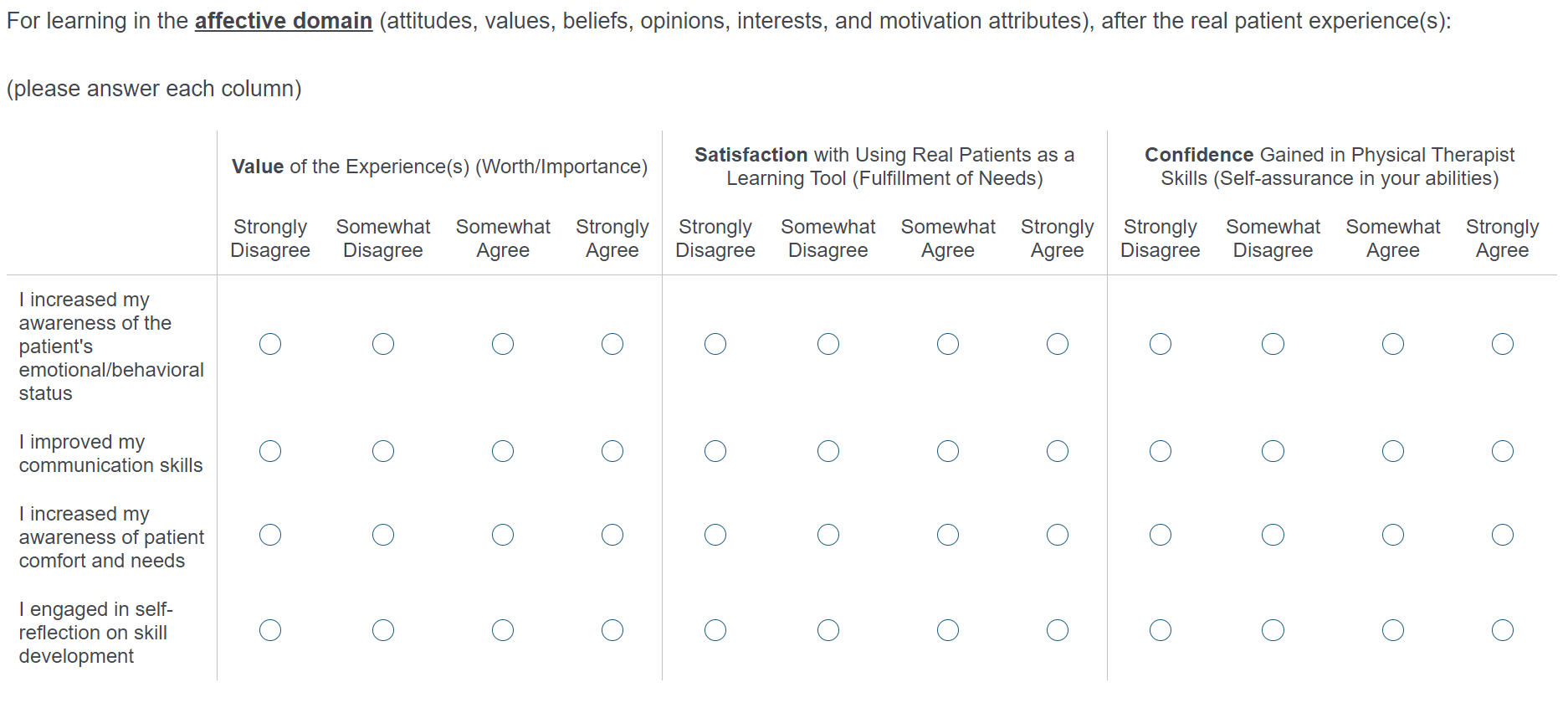


End of block: block 8 | start of block: demographics

**Q6** Which best describes the type of institution you attend?

▼ Public (1) ... Unknown (3)

**Q7** Which best describes your current year in physical therapy school?

▼ 1st year (1) ... 4th year

**Q8** What is your age?

▼ 20 (1) ... Prefer not to say

*Display this question: If what is your age? = 51+*

**Q8a** If older than 51 years of age, please write in your age in the text box.

________________________________________________________________

**Q9** To the best of your recollection, please list each class where you had an experience with a real patient. A real patient is defined as a person with impairments due to natural causes or an individual presenting without impairments presenting typically (i.e., typically developing).

Please include classes where real patients presented normally (example: “baby lab”) or presented with impairments.

________________________________________________________________

End of block: demographics | start of block: block 7

This concludes the survey phase of the study, and your participation is greatly appreciated. An additional opportunity for your participation includes interviews with students who are willing to participate in an interview (approximately 45–60 minutes long) exploring your individual experiences with “real patients” in your PT curriculum.

Participants in the interview will receive a $25.00 gift card to Giftogram.com.

Would you be willing to participate in an interview about your experiences working with “real patients” (scheduled at a mutually convenient time)?

O Yes, I would be willing to be interviewed regarding my experiences with “real patients.”

O No, I am unable/unwilling to participate in an interview about my experiences with “real patients.”

Skip to: End of survey If this concludes the survey phase of the study, your participation is greatly appreciated. An a... = No, I am unable/unwilling to participate in an interview about my experiences with “real patients.”

Please enter your name and email address. Thank you.

End of block: block 7
