# Supplementary material for: Development and validation of a measurement tool to assess student perceptions of using real patients in physical therapy education at the Rocky Mountain University, the United States: a methodological study
Source: J Educ Eval Health Prof. 2024 Nov 7;21:30. doi: 10.3352/jeehp.2024.21.30 (PMC11637597; doi:10.3352/jeehp.2024.21.30)
Supplement: Supplementary file 4 — Supplement 3. Revised 48-item survey exploratory factor analysis loading. [file jeehp-21-30-suppl3.docx]

**Supplement 3.** Revised 48-item survey exploratory factor analysis factor loadings

**Factor 1**

| Question | Matrix | Question text | Construct | Additional factor loading | Communality |
| --- | --- | --- | --- | --- | --- |
| 4 | Overall learning | Provided feedback | Value | 2 | 0.56 |
| 37 | Affective | Increased awareness of patient’s behavior | Value |  | 0.64 |
| 38 | Affective | Improved communication | Value | 7 | 0.83 |
| 39 | Affective | Increased awareness of patient needs | Value | 5 | 0.72 |
| 40 | Affective | Engaged in self-reflection | Value | 5, 8 | 0.73 |
| 41 | Affective | Increased awareness of patient’s behavior | Satisfaction |  | 0.83 |
| 42 | Affective | Improved communication | Satisfaction | 3, 7 | 0.87 |
| 43 | Affective | Increased awareness of patient needs | Satisfaction | 6 | 0.74 |
| 44 | Affective | Engaged in self-reflection | Satisfaction | 7 | 0.83 |
| 45 | Affective | Increased awareness of patient’s behavior | Confidence |  | 0.86 |
| 46 | Affective | Improved communication | Confidence | 7 | 0.82 |
| 47 | Affective | Increased awareness of patient needs | Confidence |  | 0.71 |
| 48 | Affective | Engaged in self-reflection | Confidence | 8 | 0.86 |

**Factor 2**

| Question | Matrix | Question text | Construct | Additional factor loading | Communality |
| --- | --- | --- | --- | --- | --- |
| 16 | Cognitive | Solidify lecture material | Value |  | 0.67 |
| 18 | Cognitive | Improved clinical decision making | Value | 9 | 0.51 |
| 19 | Cognitive | Improved safety awareness | Value |  | 0.78 |
| 20 | Cognitive | Solidify lecture material | Satisfaction |  | 0.74 |
| 21 | Cognitive | Solidify lab material | Satisfaction |  | 0.85 |
| 22 | Cognitive | Improved clinical decision making | Satisfaction | 9 | 0.74 |
| 23 | Cognitive | Improved safety awareness | Satisfaction | 9 | 0.78 |
| 24 | Cognitive | Solidify lecture material | Confidence | 4 | 0.61 |
| 25 | Cognitive | Solidify lab material | Confidence | 3 | 0.77 |

**Factor 3**

| Question | Matrix | Question text | Construct | Additional factor loading | Communality |
| --- | --- | --- | --- | --- | --- |
| 3 | Overall learning | Prepare for clinic | Value | 6 | 0.71 |
| 4 | Overall learning | Provided feedback | Value | 1 | 0.56 |
| 11 | Overall learning | Effective for learning | Confidence | 6 | 0.70 |
| 14 | Overall learning | Provided feedback | Confidence | 4 | 0.53 |
| 15 | Overall learning | Encouraged practical application of skills | Confidence | 6 | 0.72 |
| 25 | Cognitive | Solidify lab material | Confidence | 2 | 0.77 |
| 28 | Psychomotor | Patient handling skills | Value | 5, 9 | 0.72 |
| 29 | Psychomotor | Improved exam skills | Value |  | 0.73 |
| 30 | Psychomotor | Improved intervention skills | Value |  | 0.76 |
| 31 | Psychomotor | Patient handling skills | Satisfaction | 9 | 0.70 |
| 32 | Psychomotor | Improved exam skills | Satisfaction |  | 0.80 |
| 33 | Psychomotor | Improved intervention skills | Satisfaction |  | 0.75 |
| 34 | Psychomotor | Patient handling skills | Confidence |  | 0.71 |
| 35 | Psychomotor | Improved exam skills | Confidence |  | 0.78 |
| 36 | Psychomotor | Improved intervention skills | Confidence |  | 0.72 |
| 42 | Affective | Improved communication | Satisfaction | 1, 7 | 0.87 |

**Factor 4**

| Question | Matrix | Question text | Construct | Additional factor loading | Communality |
| --- | --- | --- | --- | --- | --- |
| 2 | Overall learning | Prepare for examinations | Value |  | 0.61 |
| 7 | Overall learning | Prepare for examinations | Satisfaction |  | 0.72 |
| 8 | Overall learning | Prepare for clinic | Satisfaction | 5, 6 | 0.77 |
| 9 | Overall learning | Provided feedback | Satisfaction |  | 0.63 |
| 10 | Overall learning | Encouraged practical application of skills | Satisfaction | 5, 6 | 0.73 |
| 12 | Overall learning | Prepare for examinations | Confidence | 8 | 0.68 |
| 14 | Overall learning | Provided feedback | Confidence | 5 | 0.53 |
| 24 | Cognitive | Solidify lecture material | Confidence | 2 | 0.61 |

**Factor 5**

| Question | Matrix | Question text | Construct | Additional factor loading | Communality |
| --- | --- | --- | --- | --- | --- |
| 1 | Overall learning | Effective for learning | Value |  | 0.72 |
| 6 | Overall learning | Effective for learning | Satisfaction | 6 | 0.69 |
| 8 | Overall learning | Prepare for clinic | Satisfaction | 5, 7 | 0.77 |
| 10 | Overall learning | Encouraged practical application of skills | Satisfaction | 5, 7 | 0.73 |
| 28 | Psychomotor | Patient handling skills | Value | 3, 9 | 0.72 |
| 39 | Affective | Increased awareness of patient needs | Value | 1 | 0.72 |
| 40 | Affective | Engaged in self-reflection | Value | 1, 8 | 0.73 |

**Factor 6**

| Question | Matrix | Question text | Construct | Additional factor loading | Communality |
| --- | --- | --- | --- | --- | --- |
| 3 | Overall learning | Prepare for clinic | Value | 3 | 0.71 |
| 6 | Overall learning | Effective for learning | Satisfaction | 5 | 0.69 |
| 8 | Overall learning | Prepare for clinic | Satisfaction | 4, 5 | 0.77 |
| 10 | Overall learning | Encouraged practical application of skills | Satisfaction | 4, 5 | 0.73 |
| 11 | Overall learning | Effective for learning | Confidence | 3 | 0.70 |
| 13 | Overall learning | Prepare for clinic | Confidence |  | 0.80 |
| 15 | Overall learning | Encouraged practical application of skills | Confidence | 3 | 0.72 |
| 43 | Affective | Increased awareness of patient needs | Satisfaction | 1 | 0.74 |

**Factor 7**

| Question | Matrix | Question text | Construct | Additional factor loading | Communality |
| --- | --- | --- | --- | --- | --- |
| 38 | Affective | Improved communication | Value | 1 | 0.83 |
| 42 | Affective | Improved communication | Satisfaction | 1, 3 | 0.87 |
| 46 | Affective | Improved communication | Confidence | 1 | 0.82 |

**Factor 8**

| Question | Matrix | Question text | Construct | Additional factor loading | Communality |
| --- | --- | --- | --- | --- | --- |
| 12 | Overall learning | Prepare for examinations | Confidence | 4 | 0.68 |
| 40 | Affective | Engaged in self-reflection | Value | 1, 5 | 0.73 |
| 44 | Affective | Engaged in self-reflection | Satisfaction | 1 | 0.83 |
| 48 | Affective | Engaged in self-reflection | Confidence | 1 | 0.86 |

**Factor 9**

| Question | Matrix | Question text | Construct | Additional factor loading | Communality |
| --- | --- | --- | --- | --- | --- |
| 17 | Cognitive | Solidify lab material | Value |  | 0.79 |
| 18 | Cognitive | Improved clinical decision making | Value | 2 | 0.51 |
| 22 | Cognitive | Improved clinical decision making | Satisfaction | 2 | 0.74 |
| 23 | Cognitive | Improved safety awareness | Satisfaction | 2 | 0.78 |
| 26 | Cognitive | Improved clinical decision making | Confidence |  | 0.62 |
| 27 | Cognitive | Improved safety awareness | Confidence |  | 0.60 |
| 28 | Psychomotor | Patient handling skills | Value | 3, 5 | 0.72 |
| 31 | Psychomotor | Patient handling skills | Satisfaction | 3 | 0.70 |
