# Supplementary material for: Development and validation of a measurement tool to assess student perceptions of using real patients in physical therapy education at the Rocky Mountain University, the United States: a methodological study
Source: J Educ Eval Health Prof. 2024 Nov 7;21:30. doi: 10.3352/jeehp.2024.21.30 (PMC11637597; doi:10.3352/jeehp.2024.21.30)
Supplement: Supplementary file 5 — Supplement 4. Exploratory factor analysis results for the overall learning matrix. [file jeehp-21-30-suppl4.docx]

**Supplement 4.** Exploratory factor analysis results for the overall learning matrix

**Initial exploratory factor analysis (EFA) for overall learning matrix (15 items)**

| Question | Question text | Construct | Factor 1 | Factor 2 | Factor 3 | Communality |
| --- | --- | --- | --- | --- | --- | --- |
| 1 | Effective for learning | Value | -0.60 |  |  | 0.43 |
| 2 | Prepare for examinations | Value |  | -0.64 |  | 0.53 |
| 3 | Prepare for clinic | Value | -0.70 |  | -0.38 | 0.68 |
| 4 | Provided feedback | Value | -0.32 |  | -0.66 | 0.60 |
| 5 | Encouraged practical application of skills | Value |  |  | -0.39 | 0.27 |
| 6 | Effective for learning | Satisfaction | -0.74 |  |  | 0.63 |
| 7 | Prepare for examinations | Satisfaction |  | -0.84 |  | 0.74 |
| 8 | Prepare for clinic | Satisfaction | -0.65 | -0.57 |  | 0.75 |
| 9 | Provided feedback | Satisfaction |  | -0.65 | -0.38 | 0.61 |
| 10 | Encouraged practical application of skills | Satisfaction | -0.60 | -0.61 |  | 0.73 |
| 11 | Effective for learning | Confidence | -0.68 |  | -0.41 | 0.63 |
| 12 | Prepare for examinations | Confidence |  | -0.53 | -0.33 | 0.48 |
| 13 | Prepare for clinic | Confidence | -0.73 |  | -0.38 | 0.70 |
| 14 | Provided feedback | Confidence |  |  | -0.90 | 0.90 |
| 15 | Encouraged practical application of skills | Confidence | -0.71 |  | -0.39 | 0.66 |

Overall learning value: item-to-item & item-to-total correlation

| Combination | r | 95% CI | No. | P-value |
| --- | --- | --- | --- | --- |
| Question 1–2 | 0.34 | 0.18–0.48 | 130 | <0.001 |
| Question 1–3 | 0.41 | 0.26–0.55 | 130 | <0.001 |
| Question 1–4 | 0.41 | 0.26–0.54 | 130 | <0.001 |
| Question 1–5 | 0.32 | 0.16–0.47 | 130 | <0.001 |
| Question 1–total value | 0.45 | 0.30–0.58 | 130 | <0.001 |
| Question 2–3 | 0.30 | 0.13–0.45 | 130 | 0.001 |
| Question 2–4 | 0.39 | 0.23–0.53 | 130 | <0.001 |
| Question 2–5 | 0.29 | 0.12–0.44 | 130 | 0.001 |
| Question 2–total value | 0.80 | 0.72–0.85 | 130 | <0.001 |
| Question 3–4 | 0.48 | 0.34–0.61 | 130 | <0.001 |
| Question 3–5 | 0.44 | 0.29–0.57 | 130 | <0.001 |
| Question 3–total value | 0.61 | 0.49–0.71 | 130 | <0.001 |
| Question 4–5 | 0.37 | 0.22–0.51 | 130 | <0.001 |
| Question 4–total value | 0.78 | 0.70–0.84 | 130 | <0.001 |
| Question 5–total value | 0.46 | 0.32–0.59 | 130 | <0.001 |

CI, confidence interval.

**Overall learning satisfaction: item-to-item & item-to-total correlation**

| Combination | r | 95% CI | No. | P-value |
| --- | --- | --- | --- | --- |
| Question 1–4 | 0.47 | 0.32–0.59 | 130 | <0.001 |
| Question 1–3 | 0.50 | 0.36–0.62 | 130 | <0.001 |
| Question 1–2 | 0.27 | 0.10–0.42 | 130 | 0.002 |
| Question 1–5 | 0.47 | 0.33–0.60 | 130 | <0.001 |
| Question 1–total satisfaction | 0.57 | 0.44–0.68 | 130 | <0.001 |
| Question 4–3 | 0.43 | 0.27–0.56 | 130 | <0.001 |
| Question 4–2 | 0.52 | 0.38–0.64 | 130 | <0.001 |
| Question 4–5 | 0.40 | 0.25–0.54 | 130 | <0.001 |
| Question 4–total satisfaction | 0.81 | 0.74–0.86 | 130 | <0.001 |
| Question 3–2 | 0.37 | 0.21–0.51 | 130 | <0.001 |
| Question 3–5 | 0.64 | 0.53–0.73 | 130 | <0.001 |
| Question 3–total satisfaction | 0.62 | 0.50–0.72 | 130 | <0.001 |
| Question 2–5 | 0.43 | 0.28–0.56 | 130 | <0.001 |
| Question 2–total satisfaction | 0.79 | 0.71–0.84 | 130 | <0.001 |
| Question 5–total satisfaction | 0.63 | 0.51–0.72 | 130 | <0.001 |

CI, confidence interval.

**Overall learning confidence: item-to-item & item-to-total correlation**

| Combination | r | 95% CI | No. | P-value |
| --- | --- | --- | --- | --- |
| Question 1–5 | 0.38 | 0.22–0.52 | 130 | <0.001 |
| Question 1–2 | 0.56 | 0.42–0.66 | 130 | <0.001 |
| Question 1–4 | 0.53 | 0.39–0.64 | 130 | <0.001 |
| Question 1–3 | 0.61 | 0.49–0.71 | 130 | <0.001 |
| Question 1–total confidence | 0.71 | 0.61–0.78 | 130 | <0.001 |
| Question 2–5 | 0.44 | 0.29–0.57 | 130 | <0.001 |
| Question 2–4 | 0.48 | 0.33–0.60 | 130 | <0.001 |
| Question 2–3 | 0.43 | 0.27–0.56 | 130 | <0.001 |
| Question 2–total confidence | 0.78 | 0.70–0.84 | 130 | <0.001 |
| Question 5–4 | 0.57 | 0.44–0.67 | 130 | <0.001 |
| Question 5–3 | 0.62 | 0.50–0.72 | 130 | <0.001 |
| Question 5–total confidence | 0.72 | 0.62–0.79 | 130 | <0.001 |
| Question 4–3 | 0.47 | 0.33–0.60 | 130 | <0.001 |
| Question 4–total confidence | 0.82 | 0.75–0.87 | 130 | <0.001 |
| Question 3–total confidence | 0.68 | 0.58–0.77 | 130 | <0.001 |

CI, confidence interval.

**Overall learning matrix EFA with items 2 and 4 removed (9 items)**

| Variable | Factor 1 | Communality |
| --- | --- | --- |
| 1 | -0.71 | 0.51 |
| 2 | -0.78 | 0.62 |
| 3 | -0.84 | 0.70 |
| 4 | -0.78 | 0.61 |
| 5 | -0.89 | 0.80 |
| 6 | -0.86 | 0.73 |
| 7 | -0.80 | 0.63 |
| 8 | -0.79 | 0.63 |
