# Supplementary material for: Development and validation of a measurement tool to assess student perceptions of using real patients in physical therapy education at the Rocky Mountain University, the United States: a methodological study
Source: J Educ Eval Health Prof. 2024 Nov 7;21:30. doi: 10.3352/jeehp.2024.21.30 (PMC11637597; doi:10.3352/jeehp.2024.21.30)
Supplement: Supplementary file 6 — Supplement 5. Exploratory factor analysis results for the cognitive matrix. [file jeehp-21-30-suppl5.docx]

**Supplement 5.** Exploratory factor analysis results for the cognitive matrix

**Cognitive matrix initial exploratory factor analysis (EFA) (12 items)**

| Variable | Factor 1 | Factor 2 | Communality |
| --- | --- | --- | --- |
| 1 | -0.76 |  | 0.64 |
| 2 | -0.73 |  | 0.63 |
| 3 | -0.37 | -0.55 | 0.43 |
| 4 |  | -0.82 | 0.71 |
| 5 | -0.78 | -0.34 | 0.73 |
| 6 | -0.76 | -0.37 | 0.71 |
| 7 | -0.51 | -0.65 | 0.68 |
| 8 |  | -0.80 | 0.71 |
| 9 | -0.74 |  | 0.63 |
| 10 | -0.75 | -0.36 | 0.70 |
| 11 | -0.37 | -0.61 | 0.51 |
| 12 |  | -0.65 | 0.48 |

**Cognitive value: item-to-item & item-to-total correlation**

| Combination | r | 95.00% CI | No. | P-value |
| --- | --- | --- | --- | --- |
| Question 1–4 | 0.24 | 0.07–0.39 | 130 | 0.007 |
| Question 1–3 | 0.36 | 0.20–0.50 | 130 | <0.001 |
| Question 1–2 | 0.52 | 0.38–0.64 | 130 | <0.001 |
| Question 1–total value | 0.78 | 0.70–0.84 | 130 | <0.001 |
| Question 4–3 | 0.37 | 0.21–0.51 | 130 | <0.001 |
| Question 4–2 | 0.31 | 0.15–0.46 | 130 | <0.001 |
| Question 4–total value | 0.53 | 0.40–0.65 | 130 | <0.001 |
| Question 3–2 | 0.31 | 0.15–0.46 | 130 | <0.001 |
| Question 3–total value | 0.70 | 0.60–0.78 | 130 | <0.001 |
| Question 2–total value | 0.70 | 0.60–0.78 | 130 | <0.001 |

CI, confidence interval.

**Cognitive satisfaction: item-to-item & item-to-total correlation**

| Combination | r | 95.00% CI | No. | P-value |
| --- | --- | --- | --- | --- |
| Question 1–2 | 0.65 | 0.54–0.74 | 130 | <0.001 |
| Question 1–3 | 0.56 | 0.43–0.67 | 130 | <0.001 |
| Question 1–total satisfaction | 0.84 | 0.78–0.88 | 130 | <0.001 |
| Question 1–4 | 0.40 | 0.24–0.53 | 130 | <0.001 |
| Question 2–3 | 0.51 | 0.37–0.63 | 130 | <0.001 |
| Question 2–total satisfaction | 0.78 | 0.71–0.84 | 130 | <0.001 |
| Question 2–4 | 0.44 | 0.29–0.57 | 130 | <0.001 |
| Question 3–total satisfaction | 0.80 | 0.72–0.85 | 130 | <0.001 |
| Question 3–4 | 0.52 | 0.39–0.64 | 130 | <0.001 |
| Question 4–total satisfaction | 0.66 | 0.54–0.74 | 130 | <0.001 |

CI, confidence interval.

**Cognitive confidence: item-to-item & item-to-total correlation**

| Combination | r | 95% CI | No. | P-value |
| --- | --- | --- | --- | --- |
| Question 1–2 | 0.76 | 0.67–0.82 | 130 | <0.001 |
| Question 1–total confidence | 0.82 | 0.75–0.87 | 130 | <0.001 |
| Question 1–4 | 0.35 | 0.19–0.49 | 130 | <0.001 |
| Question 1–3 | 0.51 | 0.37–0.63 | 130 | <0.001 |
| Question 2–total confidence | 0.85 | 0.79–0.89 | 130 | <0.001 |
| Question 2–4 | 0.47 | 0.33–0.60 | 130 | <0.001 |
| Question 2–3 | 0.56 | 0.43–0.67 | 130 | <0.001 |
| Question 4–total confidence | 0.68 | 0.57–0.76 | 130 | <0.001 |
| Question 3–total confidence | 0.81 | 0.74–0.86 | 130 | <0.001 |
| Question 3–4 | 0.61 | 0.49–0.71 | 130 | <0.001 |

CI, confidence interval.

**Cognitive matrix EFA with questions 3 and 4 removed (6 items)**

| Variable | Factor 1 | Communality |
| --- | --- | --- |
| 1 | -0.80 | 0.65 |
| 2 | -0.80 | 0.64 |
| 3 | -0.85 | 0.72 |
| 4 | -0.85 | 0.72 |
| 5 | -0.79 | 0.63 |
| 6 | -0.83 | 0.69 |
