# Supplementary material for: Development and validation of a measurement tool to assess student perceptions of using real patients in physical therapy education at the Rocky Mountain University, the United States: a methodological study
Source: J Educ Eval Health Prof. 2024 Nov 7;21:30. doi: 10.3352/jeehp.2024.21.30 (PMC11637597; doi:10.3352/jeehp.2024.21.30)
Supplement: Supplementary file 7 — Supplement 6. Exploratory factor analysis results for the psychomotor matrix. [file jeehp-21-30-suppl6.docx]

**Supplement 6.** Exploratory factor analysis results for the psychomotor matrix

**Psychomotor matrix exploratory factor analysis (EFA) (9 items)**

| Variable | Factor 1 | Communality |
| --- | --- | --- |
| 1 | -0.71 | 0.51 |
| 2 | -0.78 | 0.62 |
| 3 | -0.84 | 0.70 |
| 4 | -0.78 | 0.61 |
| 5 | -0.89 | 0.80 |
| 6 | -0.86 | 0.73 |
| 7 | -0.80 | 0.63 |
| 8 | -0.79 | 0.63 |
| 9 | -0.80 | 0.64 |

**Psychomotor value: item-to-item & item-to-total correlation**

| Combination | r | 95% CI | No. | P-value |
| --- | --- | --- | --- | --- |
| Question 1–2 | 0.64 | 0.52–0.73 | 130 | <0.001 |
| Question 1–total value | 0.67 | 0.57–0.76 | 130 | <0.001 |
| Question 1–3 | 0.58 | 0.45–0.68 | 130 | <0.001 |
| Question 2–total value | 0.87 | 0.82–0.91 | 130 | <0.001 |
| Question 2–3 | 0.67 | 0.56–0.75 | 130 | <0.001 |
| Question 3–total value | 0.91 | 0.88–0.94 | 130 | <0.001 |

CI, confidence interval.

**Psychomotor satisfaction: item-to-item & item-to-total correlation**

| Combination | r | 95% CI | No. | P-value |
| --- | --- | --- | --- | --- |
| Question 1–3 | 0.64 | 0.53–0.73 | 130 | <0.001 |
| Question 1–total satisfaction | 0.75 | 0.66–0.81 | 130 | <0.001 |
| Question 1–2 | 0.63 | 0.52–0.73 | 130 | <0.001 |
| Question 3–total satisfaction | 0.89 | 0.85–0.92 | 130 | <0.001 |
| Question 3–2 | 0.74 | 0.65–0.81 | 130 | <0.001 |
| Question 2–total satisfaction | 0.90 | 0.87–0.93 | 130 | <0.001 |

CI, confidence interval.

**Psychomotor confidence: item-to-item & item-to-total correlation**

| Combination | r | 95% CI | No. | P-value |
| --- | --- | --- | --- | --- |
| Question 1–2 | 0.65 | 0.53–0.74 | 130 | <0.001 |
| Question 1–total confidence | 0.78 | 0.71–0.84 | 130 | <0.001 |
| Question 1–3 | 0.64 | 0.53–0.73 | 130 | <0.001 |
| Question 2–total confidence | 0.88 | 0.83–0.91 | 130 | <0.001 |
| Question 2–3 | 0.72 | 0.63–0.80 | 130 | <0.001 |
| Question 3–total confidence | 0.91 | 0.87–0.94 | 130 | <0.001 |

CI, confidence interval.
