# Supplementary material for: Development and validation of a measurement tool to assess student perceptions of using real patients in physical therapy education at the Rocky Mountain University, the United States: a methodological study
Source: J Educ Eval Health Prof. 2024 Nov 7;21:30. doi: 10.3352/jeehp.2024.21.30 (PMC11637597; doi:10.3352/jeehp.2024.21.30)
Supplement: Supplementary file 8 — Supplement 7. Exploratory factor analysis results for the affective matrix. [file jeehp-21-30-suppl7.docx]

**Supplement 7.** Exploratory factor analysis results for the affective matrix

**Affective matrix initial exploratory factor analysis (EFA) (12 items)**

| Variable | Factor 1 | Factor 2 | Communality |
| --- | --- | --- | --- |
| 1 | -0.54 | -0.42 | 0.47 |
| 2 |  | -0.84 | 0.73 |
| 3 | -0.57 | -0.54 | 0.61 |
| 4 | -0.80 |  | 0.66 |
| 5 | -0.69 | -0.51 | 0.73 |
| 6 |  | -0.89 | 0.85 |
| 7 | -0.54 | -0.64 | 0.70 |
| 8 | -0.84 |  | 0.75 |
| 9 | -0.73 | -0.48 | 0.76 |
| 10 | -0.32 | -0.81 | 0.75 |
| 11 | -0.64 | -0.49 | 0.66 |
| 12 | -0.77 |  | 0.63 |

**Affective value: item-to-item & item-to-total correlation**

| Combination | r | 95% CI | No. | P-value |
| --- | --- | --- | --- | --- |
| Question 1–4 | 0.48 | 0.33–0.60 | 130 | <0.001 |
| Question 1–2 | 0.36 | 0.20–0.50 | 130 | <0.001 |
| Question 1–3 | 0.54 | 0.41–0.65 | 130 | <0.001 |
| Question 1–total value | 0.75 | 0.66–0.82 | 130 | <0.001 |
| Question 4–2 | 0.26 | 0.09–0.41 | 130 | 0.003 |
| Question 4–3 | 0.49 | 0.34–0.61 | 130 | <0.001 |
| Question 4–total value | 0.71 | 0.62–0.79 | 130 | <0.001 |
| Question 2–3 | 0.53 | 0.39–0.64 | 130 | <0.001 |
| Question 2–total value | 0.69 | 0.59–0.77 | 130 | <0.001 |
| Question 3–total value | 0.73 | 0.64–0.80 | 130 | <0.001 |

CI, confidence interval.

**Affective satisfaction: item-to-item & item-to-total correlation**

| Combination | r | 95% CI | No. | P-value |
| --- | --- | --- | --- | --- |
| Question 1–4 | 0.60 | 0.48–0.70 | 130 | <0.001 |
| Question 1–3 | 0.68 | 0.58–0.76 | 130 | <0.001 |
| Question 1–2 | 0.64 | 0.52–0.73 | 130 | <0.001 |
| Question 1–total satisfaction | 0.84 | 0.78–0.89 | 130 | <0.001 |
| Question 4–3 | 0.56 | 0.43–0.67 | 130 | <0.001 |
| Question 4–2 | 0.49 | 0.35–0.61 | 130 | <0.001 |
| Question 4–total satisfaction | 0.80 | 0.73–0.85 | 130 | <0.001 |
| Question 3–2 | 0.69 | 0.58–0.77 | 130 | <0.001 |
| Question 3–total satisfaction | 0.83 | 0.77–0.88 | 130 | <0.001 |
| Question 2–total satisfaction | 0.80 | 0.73–0.86 | 130 | <0.001 |

CI, confidence interval.

**Affective confidence: item-to-item & item-to-total correlation**

| Combination | r | 95% CI | No. | P-value |
| --- | --- | --- | --- | --- |
| Question 1–4 | 0.70 | 0.60–0.78 | 130 | <0.001 |
| Question 1–3 | 0.73 | 0.64–0.80 | 130 | <0.001 |
| Question 1–2 | 0.63 | 0.51–0.72 | 130 | <0.001 |
| Question 1–total confidence | 0.87 | 0.83–0.91 | 130 | <0.001 |
| Question 4–3 | 0.57 | 0.44–0.68 | 130 | <0.001 |
| Question 4–2 | 0.53 | 0.39–0.64 | 130 | <0.001 |
| Question 4–total confidence | 0.83 | 0.76–0.87 | 130 | <0.001 |
| Question 3–2 | 0.61 | 0.49–0.71 | 130 | <0.001 |
| Question 3–total confidence | 0.83 | 0.76–0.87 | 130 | <0.001 |
| Question 2–total confidence | 0.80 | 0.73–0.86 | 130 | <0.001 |

CI, confidence interval.

**Affective matrix with questions 3 and 4 removed (6 items)**

| Variable | Factor 1 | Communality |
| --- | --- | --- |
| 1 | -0.74 | 0.55 |
| 2 | -0.80 | 0.65 |
| 3 | -0.89 | 0.78 |
| 4 | -0.84 | 0.71 |
| 5 | -0.88 | 0.77 |
| 6 | -0.85 | 0.72 |
