# Supplementary material for: Development and validation of a measurement tool to assess student perceptions of using real patients in physical therapy education at the Rocky Mountain University, the United States: a methodological study
Source: J Educ Eval Health Prof. 2024 Nov 7;21:30. doi: 10.3352/jeehp.2024.21.30 (PMC11637597; doi:10.3352/jeehp.2024.21.30)
Supplement: Supplementary file 9 — Supplement 8. Revised survey matrices (30-item survey). [file jeehp-21-30-suppl8.docx]

**Supplement 8.** Revised survey matrices (30-item survey)

**Overall learning matrix**

For your **overall learning**, experience(s) with real patients: (please answer each column)

|  | **Value** of the experience(s) (worth/importance) | | | | **Satisfaction** with using real patients as a learning tool (fulfillment of needs) | | | | **Confidence** gained in physical therapist skills (self-assurance in your abilities) | | | |
| --- | --- | --- | --- | --- | --- | --- | --- | --- | --- | --- | --- | --- |
|  | Strongly disagree | Somewhat disagree | Somewhat agree | Strongly agree | Strongly disagree | Somewhat disagree | Somewhat agree | Strongly agree | Strongly disagree | Somewhat disagree | Somewhat agree | Strongly agree |
| Was effective for my learning | **○** | **○** | **○** | **○** | **○** | **○** | **○** | **○** | **○** | **○** | **○** | **○** |
| Prepared me for the setting | **○** | **○** | **○** | **○** | **○** | **○** | **○** | **○** | **○** | **○** | **○** | **○** |
| Encouraged practical application of physical therapist skills | **○** | **○** | **○** | **○** | **○** | **○** | **○** | **○** | **○** | **○** | **○** | **○** |

**Cognitive matrix**

For learning in the **cognitive domain** (facts and knowledge needed to perform physical therapist duties), after the real patient experience(s): (please answer each column)

|  | **Value** of the experience(s) (worth/importance) | | | | **Satisfaction** with using real patients as a learning tool (fulfillment of needs) | | | | **Confidence** gained in physical therapist skills (self-assurance in your abilities) | | | |
| --- | --- | --- | --- | --- | --- | --- | --- | --- | --- | --- | --- | --- |
|  | Strongly disagree | Somewhat disagree | Somewhat agree | Strongly agree | Strongly disagree | Somewhat disagree | Somewhat agree | Strongly agree | Strongly disagree | Somewhat disagree | Somewhat agree | Strongly agree |
| Helped in solidifying my knowledge material | **○** | **○** | **○** | **○** | **○** | **○** | **○** | **○** | **○** | **○** | **○** | **○** |
| Helped in solidifying my knowledge of lab material | **○** | **○** | **○** | **○** | **○** | **○** | **○** | **○** | **○** | **○** | **○** | **○** |

**Psychomotor matrix**

For learning in the **psychomotor domain** (hands-on, performance-based skills), after the real patient experience(s): (please answer each column)

|  | **Value** of the experience(s) (worth/importance) | | | | **Satisfaction** with using real patients as a learning tool (fulfillment of needs) | | | | **Confidence** gained in physical therapist skills (self-assurance in your abilities) | | | |
| --- | --- | --- | --- | --- | --- | --- | --- | --- | --- | --- | --- | --- |
|  | Strongly disagree | Somewhat disagree | Somewhat agree | Strongly agree | Strongly disagree | Somewhat disagree | Somewhat agree | Strongly agree | Strongly disagree | Somewhat disagree | Somewhat agree | Strongly agree |
| I improved my patient handling skills | **○** | **○** | **○** | **○** | **○** | **○** | **○** | **○** | **○** | **○** | **○** | **○** |
| I improved my performance in examination skills | **○** | **○** | **○** | **○** | **○** | **○** | **○** | **○** | **○** | **○** | **○** | **○** |
| I improved my performance in intervention skills | **○** | **○** | **○** | **○** | **○** | **○** | **○** | **○** | **○** | **○** | **○** | **○** |

**Affective matrix**

For learning in the **affective domain** (attitudes, values, beliefs, opinions, interests, and motivation attributes), after the real patient experience(s): (please answer each column)

|  | **Value** of the experience(s) (worth/importance) | | | | **Satisfaction** with using real patients as a learning tool (fulfillment of needs) | | | | **Confidence** gained in physical therapist skills (self-assurance in your abilities) | | | |
| --- | --- | --- | --- | --- | --- | --- | --- | --- | --- | --- | --- | --- |
|  | Strongly disagree | Somewhat disagree | Somewhat agree | Strongly agree | Strongly disagree | Somewhat disagree | Somewhat agree | Strongly agree | Strongly disagree | Somewhat disagree | Somewhat agree | Strongly agree |
| I increased my awareness of the patient’s emotional/behavioral status | **○** | **○** | **○** | **○** | **○** | **○** | **○** | **○** | **○** | **○** | **○** | **○** |
| I increased my awareness of patient comfort and needs | **○** | **○** | **○** | **○** | **○** | **○** | **○** | **○** | **○** | **○** | **○** | **○** |
