# Supplementary material for: Development and validation of a measurement tool to assess student perceptions of using real patients in physical therapy education at the Rocky Mountain University, the United States: a methodological study
Source: J Educ Eval Health Prof. 2024 Nov 7;21:30. doi: 10.3352/jeehp.2024.21.30 (PMC11637597; doi:10.3352/jeehp.2024.21.30)
Supplement: Supplementary file 10 — Supplement 9. 30-item survey final results, including exploratory factor analysis, content validity index, Cronbach’s α. [file jeehp-21-30-suppl9.docx]

**Supplement 9.** 30-item survey final results, including exploratory factor analysis, content validity index, Cronbach’s α

**Final Cronbach’s α, exploratory factor analysis (EFA), and content validity index (CVI)**

| Matrix domains and items | Cronbach’s α | EFA loadings | Extraneous factor loadings | CVI |
| --- | --- | --- | --- | --- |
| **Overall learning** |  |  |  | 1.0 |
| Value | 0.65 |  |  |  |
| • Was effective for my learning. |  | -0.44 | 0.64 |  |
| • Prepare me for the clinical setting. |  | -0.73 |  |  |
| • Encouraged practical application of physical therapist skills. |  |  | -0.34 |  |
| Satisfaction | 0.85 |  |  |  |
| • Was effective for my learning. |  | -0.60 | 0.47 |  |
| • Prepare me for the clinical setting. |  | -0.69 |  |  |
| • Encouraged practical application of physical therapist skills. |  | -0.66 |  |  |
| Confidence | 0.87 |  |  |  |
| • Was effective for my learning. |  | -0.60 | -0.36 |  |
| • Prepare me for the clinical setting. |  | -0.83 |  |  |
| • Encouraged practical application of physical therapist skills. |  | -0.59 | -0.37 |  |
| **Cognitive** |  |  |  | 1.0 |
| Value | 0.78 |  |  |  |
| • Helped solidifying my knowledge of lecture material. |  | -0.84 |  |  |
| • Help solidifying my knowledge of lab material. |  | -0.74 |  |  |
| Satisfaction | 0.81 |  |  |  |
| • Helped solidifying my knowledge of lecture material. |  | -0.74 |  |  |
| • Help solidifying my knowledge of lab material. |  | -0.85 |  |  |
| Confidence | 0.87 |  |  |  |
| • Helped solidifying my knowledge of lecture material. |  | -0.67 |  |  |
| • Help solidifying my knowledge of lab material. |  | -0.71 | -0.40 |  |
| **Psychomotor** |  |  |  | 1.0 |
| Value | 0.86 |  |  |  |
| • I improved my patient-handling skills. |  | -0.56 |  |  |
| • I improved my performance in examination skills. |  | -0.68 |  |  |
| • I improved my performance in intervention skills. |  | -0.74 |  |  |
| Satisfaction |  |  |  |  |
| • I improved my patient-handling skills. | 0.89 | -0.63 |  |  |
| • I improved my performance in examination skills. |  | -0.77 |  |  |
| • I improved my performance in intervention skills. |  | -0.79 |  |  |
| Confidence | 0.87 |  |  |  |
| • I improved my patient-handling skills. |  | -0.80 |  |  |
| • I improved my performance in examination skills. |  | -0.81 |  |  |
| • I improved my performance in intervention skills. |  | -0.90 |  |  |
| **Affective** |  |  |  | 1.0 |
| Value | 0.76 |  |  |  |
| • I increased my awareness of the patient’s emotional/behavioral status. |  | 0.70 |  |  |
| • I increased my awareness of patient comfort and needs. |  | 0.69 |  |  |
| Satisfaction | 0.85 |  |  |  |
| • I increased my awareness of the patient’s emotional/behavioral status. |  | 0.84 |  |  |
| • I increased my awareness of patient comfort and needs. |  | 0.72 | -0.33 |  |
| Confidence | 0.93 |  |  |  |
| • I increased my awareness of the patient’s emotional/behavioral status. |  | 0.85 |  |  |
| • I increased my awareness of patient comfort and needs. |  | 0.73 | -0.32 |  |
